# Incorporation of clinical and biological factors improves prognostication and reflects contemporary clinical practice

**DOI:** 10.1038/s41523-020-0152-4

**Published:** 2020-03-25

**Authors:** Rashmi K. Murthy, Juhee Song, Akshara S. Raghavendra, Yisheng Li, Limin Hsu, Kenneth R. Hess, Carlos H. Barcenas, Vicente Valero, Robert W. Carlson, Debu Tripathy, Gabriel N. Hortobagyi

**Affiliations:** 1 Department of Breast Medical Oncology, The University of Texas MD Anderson Cancer Center, Houston, TX USA; 2 Department of Biostatistics, The University of Texas MD Anderson Cancer Center, Houston, TX USA; 3 National Comprehensive Cancer Network® (NCCN®), Fort Washington, PA USA; 4 Department of Hematology/Oncology Fox Chase Cancer Center, Philadelphia, PA USA

**Keywords:** Breast cancer, Breast cancer

## Abstract

We developed prognostic models for breast cancer-specific survival (BCSS) that consider anatomic stage and other important determinants of prognosis and survival in breast cancer, such as age, grade, and receptor-based subtypes with the intention to demonstrate that these factors, conditional on stage, improve prediction of BCSS. A total of 20,928 patients with stage I–III invasive primary breast cancer treated at The University of Texas MD Anderson Cancer Center between 1990 and 2016, who received surgery as an initial treatment were identified to generate prognostic models by Fine-Gray competing risk regression model. Model predictive accuracy was assessed using Harrell’s C-index. The Aalen–Johansen estimator and a selected Fine–Gray model were used to estimate the 5-year and 10-year BCSS probabilities. The performance of the selected model was evaluated by assessing discrimination and prediction calibration in an external validation dataset of 29,727 patients from the National Comprehensive Cancer Network (NCCN). The inclusion of age, grade, and receptor-based subtype in addition to stage significantly improved the model predictive accuracy (C-index: 0.774 (95% CI 0.755–0.794) vs. 0.692 for stage alone, *p* < 0.0001). Young age (<40), higher grade, and TNBC subtype were significantly associated with worse BCSS. The selected model showed good discriminative ability but poor calibration when applied to the validation data. After recalibration, the predictions showed good calibration in the training and validation data. More refined BCSS prediction is possible through a model that has been externally validated and includes clinical and biological factors.

## Introduction

Progress in breast cancer biology, research, and treatment has made the incorporation of clinical and pathological characteristics into the staging system necessary. The American Joint Committee on Cancer (AJCC) TNM staging classification is globally accepted to describe extent of cancer at diagnosis and has been available since 1959. The AJCC has incorporated biological factors (e.g. Gleason’s score, mitotic index, and grade) into the staging classification of cancers such as prostate, melanoma, and sarcoma^1–3^. In contrast, until recently, the breast cancer TNM classification has had only anatomical-based staging criteria without the addition of biological, prognostic, and predictive factors used to guide treatment in the clinical practice^4–8^. It is evident that a staging system based solely on anatomy, does not always reflect the variable clinical course and long-term outcomes seen in clinical experience^9^. Age, tumor grade, hormone receptor (HR) status, and human epidermal growth factor receptor-2 (HER2) status are well-recognized prognostic factors and the latter two also serve as predictors of response to endocrine and anti-HER2 therapy, respectively^10–29^. Prior work with robust statistical approaches has indicated the need to include such factors in a refined staging system^30–33^.

The recently published AJCC 8th edition recognizes new prognostic categories that significantly improve prognostic categorization compared to the anatomic stage groupings alone^34^. To date, there are no robust breast cancer outcome prediction tools available to practicing clinicians to provide patients with early stage breast cancer survival estimates based on the presenting features of their tumor and other clinical factors. Prior tools have been developed but have used different statistical approaches, i.e. mathematical modeling, Cox proportion hazards regression, or actuarial analysis; lacking in follow-up time; more focused on guiding adjuvant therapy discussions; or do not represent a contemporary patient population^35–38^. To accompany the recent update to the AJCC staging system, we sought in this analysis, to develop a validated model and demonstrate that age, tumor grade, and biomarker subtypes, conditional on stage, were important determinants of BCSS and that their incorporation could further refine the survival estimates based upon stage utilizing a Fine–Gray Model. Further, we have developed an online tool to estimate individual prognosis based on this model combining clinical and biologic variables for use in daily clinical practice.

## Results

### Prediction model on the training data

In the MDACC database, 20,928 patients with stage I–III breast cancer, who received treatment with surgery as the first intervention were identified (see Supplementary Table 1 for the characteristics of the overall cohort). From the overall cohort (*n* = 20,928), a complete data cohort (*N* = 14,781) was formed for model comparison purposes, after excluding 29% of the patients (*n* = 6147) missing one or more of the key variables (age, HR, HER2, grade). All models with stage and additional factors showed higher C-index than the model with stage alone (Table 1). The model with age, grade, combined HR and HER2 status, and stage, which reflects the information conventionally available in current clinical practice, showed the best predictive accuracy.

**Table 1:** Comparisons of multivariable Fine–Gray models, using complete data cohort (*N* = 14,781)^a^.

Model	Covariate	C-index^b^ (95% CI)			*p*-value^c^
Model 0	Stage	0.692	0.681	0.703	–
Model 1	Stage, Age	0.701	0.689	0.712	<0.0001
Model 2	Stage, HR/Her2^d^	0.745	0.739	0.761	<0.0001
Model 3	Stage, Grade	0.757	0.747	0.767	<0.0001
Model 4	Stage, HR/Her2^d^, Age	0.752	0.741	0.763	<0.0001
Model 5	Stage, HR/Her2^d^, Grade	0.740	0.729	0.751	<0.0001
Model 6	Stage, Age, Grade	0.758	0.748	0.768	<0.0001
Model 7	Stage, Age, HR/Her2^d^, Grade	0.774	0.764	0.783	<0.0001

^a^Patients with complete data (age, stage, HR, HER2, Grade) included.

^b^Harrell’s C- Index: The kmi package in R was used to impute censoring times for competing risk data and the rcorr.cens function in the Hmisc package in R was used to estimate the C-index and its confidence interval.

^c^*p*-value comparing C-index values between models was computed using the compareC package in R.

^d^*ER* estrogen receptor, *PR* progesterone receptor, *HER2* HER2-neu receptor, *HR* hormone receptor; HR+: ER+ or PR+; HR−: ER− and PR−.

Table 2 shows the characteristics of the patients included in the multivariate analysis from the training dataset (*N* = 14,781) and validation dataset (*N* = 29,727). The median follow-up was 6.6 years (95% CI, 6.5–6.7) (range: 0–24.9 years) and the median age was 55 (range 20–99) years. The 5-year BCSS estimate was 89% for the whole multivariate cohort. Outcomes for the multivariate cohort were as follows: 15% BC-related mortality, 20% mortality from any cause, 10% loco-regional recurrence, and 20% distant recurrence (first loco-regional recurrence and distant recurrence were 8% and 16%, respectively).

**Table 2:** Patient characteristics for the multivariate Fine–Gray model: complete data cohort in training and validation datasets.

Variable	Training data (*N* = 14,781)		Validation data (*N* = 29,727)	
	*N*	%	*N*	%
Race^a^				
White	10,853	73	16,812	57
Black	1391	9	1303	4
Hispanic	1767	12	784	3
Other	770	5	785	3
Unknown	0	0	10,043	34
Age				
<40	1582	11	1913	6
40–69	11,351	77	22,151	75
≥70	1848	12	5663	19
Menopausal status^b^				
Premenopausal	5182	35	17,332	58
Postmenopausal	9599	65	12,395	42
Anatomic stage				
I	7688	52	16,607	56
IIA	3694	25	8327	28
IIB	1870	13	3331	11
IIIA–IIIB	1076	7	737	3
IIIC	453	3	725	2
Nuclear grade^c^				
1	1885	13	6723	23
2	6627	45	13,884	47
3	6269	42	9120	31
ER status^d^				
Positive	11,623	79	24,646	83
Negative	3154	21	5077	17
Unknown	4	0	4	0
PR status^d^				
Positive	9790	66	21,827	74
Negative	4944	33	7856	27
Unknown	47	0	44	0
Hormone receptor (HR) status^e^				
HR+^c^	11,888	80	24,989	84
HR−^d^	2893	20	4738	16
HER2 status^d,f^				
Positive	1175	8	1086	4
Negative	13,606	92	28,641	96
Biomarker subgroups				
TNBC	2431	16	4401	15
HR+/HER2–	11,175	76	24,240	82
HR+/HER2+^e^	713	5	749	3
HR−/HER2+	462	3	337	1
Chemotherapy^f^				
TNBC	1914	79	3459	79
HR+/HER2−	5035	45	8866	37
HR+/HER2+	657	92	748	100
HR−/HER2+	426	92	335	99

^a^For NCCN data, race background and ethnicity variables were used to obtain this variable.

^b^Clinically defined based on history; if not recorded, age is considered (≥60: postmenopausal; <60: premenopausal).

^c^Histologic grade or nuclear grade (if histologic grade is not available); Training Data, HG (*n* = 4724); NG (*n* = 10,057); Validation Data, HG (*n* = 29,466); NG (*n* = 12,434).

^d^Biomarker definitions in database are reflective of evolution of national guidelines^28,38,54–56^.

^e^HR+:ER+ or PR+; HR−: ER− and PR−.

^f^All HER2+ patients included in the multivariate analysis received adjuvant trastuzumab.

**Fig. 1:**
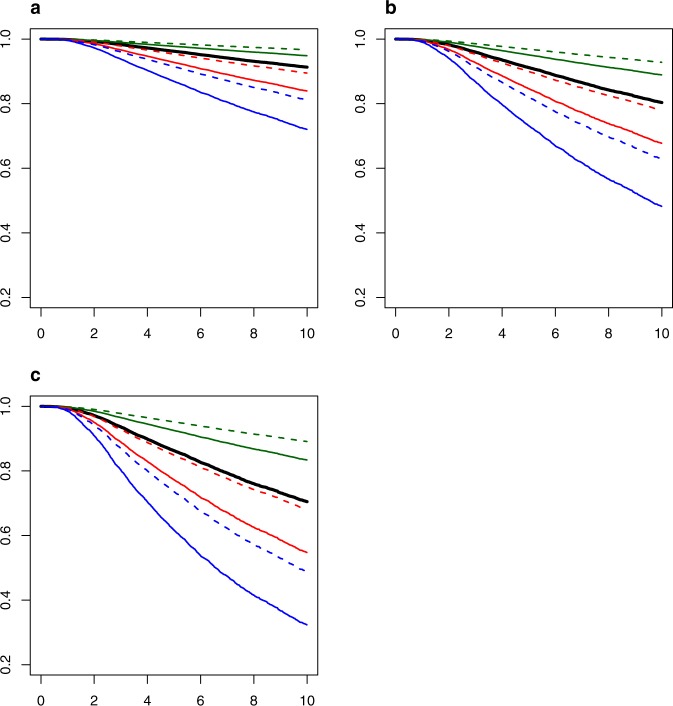
BCSS estimation. Estimated BCSS by combining age group, receptor subtype, grade within each stage group (1, 2A, 2B) based on Fine–Gray model 7 using average baseline survival (*N* = 14,781).

In multivariate analysis, age, stage, biologic subtype, and grade were associated with BCSS (Table 3). There was some indication of violations of the PH assumption, especially for HR/Her2 subtype. However, we did not explore the Fine–Gray model with time-varying covariates because the analysis would be computationally complex and the resulting model could still yield serious biases^39,40^. Despite the violation of the PH assumption, our estimated hazard ratios are still interpretable as weighted average hazard ratios over the follow-up^41^. Figure 1 shows examples for the largest stage groupings (I, IIA, IIB) demonstrating that the curves can be further refined with the combination of factors including age group, receptor-subtype, and nuclear grade within each anatomic stage group. In each stage grouping, patients aged 40–69 with a grade 1 HR+/HER2− cancers have the best survival; whereas patients with age < 40, grade 3, TNBC cancers have the worst survival. The impact of age is also clear; younger patients (<40) with a grade 3 TNBC consistently have worse survival compared to an older age group 40–69 keeping other factors constant (TNBC and grade 3).

**Table 3:** Multivariate Fine–Gray model (Model 7) (*N* = 14,781)^a^ parameter estimates based on Fine–Gray model.

Covariate	Level	Parameter estimate	SE	SHR	95% CI	*p*
Age at diagnosis	<40	0.45410	0.057	1.58	1.41–1.76	<0.0001
	40–69			REF		
	≥70	−0.18590	0.084	0.83	0.70–0.98	0.0268
Anatomic stage	I			REF		
	IIA	0.79969	0.058	2.23	1.99–2.49	<0.0001
	IIB	1.23600	0.064	3.44	3.04–3.90	<0.0001
	IIIAB	1.42681	0.077	4.17	3.58–4.84	<0.0001
	IIIC	1.85219	0.096	6.37	5.28–7.70	<0.0001
Biologic subtype	TNBC	0.62773	0.056	1.87	1.68–2.09	<0.0001
	HR+/HER2+(T)(T)	−0.38992	0.125	0.68	0.53–0.87	0.002
	HR−/HER2+(T)	0.06342	0.124	1.07	0.84–1.36	0.61
	HR+/HER2−			REF		
Grade^b^	1			REF		
	2	0.56614	0.119	1.76	1.40–2.22	<0.0001
	3	1.19932	0.121	3.32	2.62–4.21	<0.0001

SHR refers to the subdistribution hazard ratio of mortality with breast cancer.

*SHR* subdistribution hazard ratio, *CI* confidence interval, *Ref* reference group (1.00).

^a^Patients with complete data including age, stage, hormone receptor (HR) status (HR+: ER+ or PR+ and HR−: ER− and PR−), HER2 status, and grade were included.

^b^Histologic grade or nuclear grade (if histologic grade is not available); HG (*n* = 4724); NG (*n* = 10,057).

Table 4 shows refined 5-year and 10-year BCSS estimates by age group (<40: A, 40–69: B, and ≥70: C), HR status, HER2 status, and pathologic stage for each grade. For TNBC, Grade 3, age < 40, the 5-year BCSS is noted to decrease by stage: 88% (1); 76% (IIA), 65% (IIB), 60% (IIIAB), and 45% (IIIC). In contrast, for TNBC, grade 3, age 40–69, the 5-year BCSS is as follows: 92% (I), 84% (IIA), 76% (IIB), 72% (IIIAB), and 60% (IIIC). Within the age group < 40 and HR+/HER2− subtype, the 5-year BCSS within each stage group is different for grade 1 vs. grade 3 tumors: 98% vs. 94% (I), 96% vs. 86% (IIA), 93% vs. 80% (IIB), 92% vs. 76% (IIIAB), 88% vs. 66% (IIIC). Similarly, within the age group 40–69 similar contrasts are noted for HR+/HER2− by grade (1 vs. 3): 99% vs. 96% (I), 97% vs. 91% (IIA), 96% vs. 87% (IIB), 95% vs. 84% (IIIAB), and 92% vs. 76% (IIIC). The results of Shoenfeld residuals, nonlinear covariate effects and two-way covariate–covariate interaction yielded no remarkable findings. Table 4 shows the 5-year and 10-year BCSS for each combination of factors (age, anatomic stage, HR, HER2, and tumor grade).

**Table 4:** Five-year and ten-year BCSS estimates by age, HR status, HER2 status, and stage for each grade based on Fine–Gray model 7 with average baseline survival (*n* = 14,781).

Grade	Age	HR	HER2	5-year BCSS					10-year BCSS				
				Stage					Stage				
				I	IIA	IIB	IIIAB	IIIC	I	IIA	IIB	IIIAB	IIIC
1	<40	Neg	Neg	96.3	92.0	87.9	85.5	78.7	91.7	82.4	74.1	69.6	57.4
		Neg	Pos	97.9	95.4	92.9	91.5	87.3	95.2	89.6	84.3	81.4	73.0
		Pos	Neg	98.0	95.6	93.3	92.0	88.0	95.5	90.2	85.2	82.4	74.4
		Pos	Pos	98.7	97.0	95.4	94.5	91.7	96.9	93.2	89.7	87.7	81.8
	40–69	Neg	Neg	97.6	94.8	92.1	90.6	85.9	94.6	88.4	82.7	79.4	70.3
		Neg	Pos	98.7	97.0	95.4	94.5	91.7	96.9	93.3	89.8	87.7	81.9
		Pos	Neg	98.7	97.2	95.7	94.8	92.2	97.1	93.7	90.4	88.4	82.9
		Pos	Pos	99.1	98.1	97.1	96.5	94.7	98.0	95.7	93.4	92.0	88.1
	≥70	Neg	Neg	98.0	95.7	93.4	92.1	88.2	95.5	90.3	85.4	82.6	74.7
		Neg	Pos	98.9	97.5	96.2	95.4	93.1	97.4	94.4	91.4	89.7	84.7
		Pos	Neg	98.9	97.7	96.4	95.7	93.5	97.6	94.7	91.9	90.3	85.6
		Pos	Pos	99.3	98.4	97.6	97.1	95.5	98.4	96.4	94.5	93.3	90.0
2	<40	Neg	Neg	93.6	86.3	79.7	75.9	65.6	85.8	71.1	59.0	52.8	37.7
		Neg	Pos	96.3	92.0	87.9	85.5	78.7	91.7	82.4	74.1	69.6	57.4
		Pos	Neg	96.5	92.5	88.6	86.3	79.9	92.1	83.4	75.5	71.1	59.4
		Pos	Pos	97.6	94.8	92.1	90.5	85.9	94.6	88.4	82.6	79.4	70.3
	40–69	Neg	Neg	95.9	91.1	86.5	84.0	76.5	90.7	80.5	71.5	66.7	53.8
		Neg	Pos	97.6	94.8	92.1	90.5	85.9	94.6	88.4	82.7	79.4	70.3
		Pos	Neg	97.8	95.1	92.6	91.1	86.7	94.9	89.1	83.6	80.5	71.8
		Pos	Pos	98.5	96.7	94.9	93.9	90.8	96.5	92.5	88.6	86.4	79.9
	≥70	Neg	Neg	96.6	92.5	88.7	86.5	80.1	92.2	83.6	75.7	71.4	59.8
		Neg	Pos	98.0	95.7	93.4	92.1	88.1	95.5	90.3	85.4	82.6	74.6
		Pos	Neg	98.2	95.9	93.8	92.5	88.8	95.8	90.9	86.2	83.6	76.0
		Pos	Pos	98.7	97.2	95.8	94.9	92.3	97.1	93.7	90.4	88.5	83.0
3	<40	Neg	Neg	88.3	75.8	65.1	59.5	45.2	74.9	52.6	37.0	30.1	15.9
		Neg	Pos	93.2	85.4	78.4	74.5	63.7	84.9	69.4	56.8	50.5	35.1
		Pos	Neg	93.6	86.3	79.6	75.8	65.5	85.7	71.0	58.9	52.6	37.5
		Pos	Pos	95.6	90.5	85.7	82.9	75.1	90.1	79.3	69.8	64.8	51.4
	40–69	Neg	Neg	92.4	83.9	76.2	71.9	60.4	83.3	66.5	53.2	46.6	31.1
		Neg	Pos	95.6	90.5	85.7	82.9	75.1	90.1	79.3	69.9	64.8	51.5
		Pos	Neg	95.9	91.0	86.5	83.9	76.4	90.7	80.4	71.4	66.5	53.6
		Pos	Pos	97.2	93.8	90.6	88.8	83.3	93.6	86.3	79.6	75.9	65.6
	≥70	Neg	Neg	93.6	86.4	79.8	76.1	65.8	85.9	71.3	59.2	53.1	37.9
		Neg	Pos	96.3	92.0	87.9	85.6	78.8	91.7	82.5	74.2	69.7	57.6
		Pos	Neg	96.6	92.5	88.6	86.4	80.0	92.2	83.5	75.6	71.3	59.6
		Pos	Pos	97.7	94.9	92.2	90.6	86.0	94.6	88.5	82.8	79.5	70.4

### Model validation

**Fig. 2:**
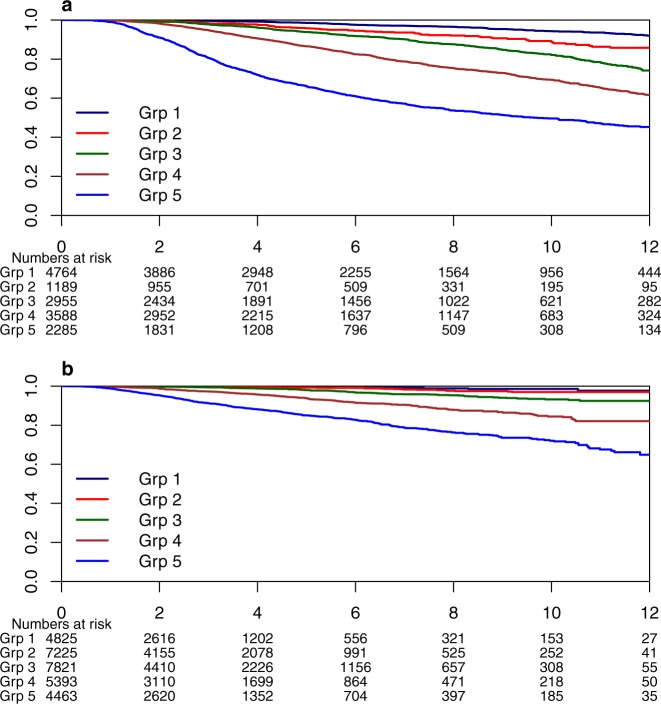
BCSS probability by risk group. **a** Training data and **b** validation data.

**Fig. 3:**
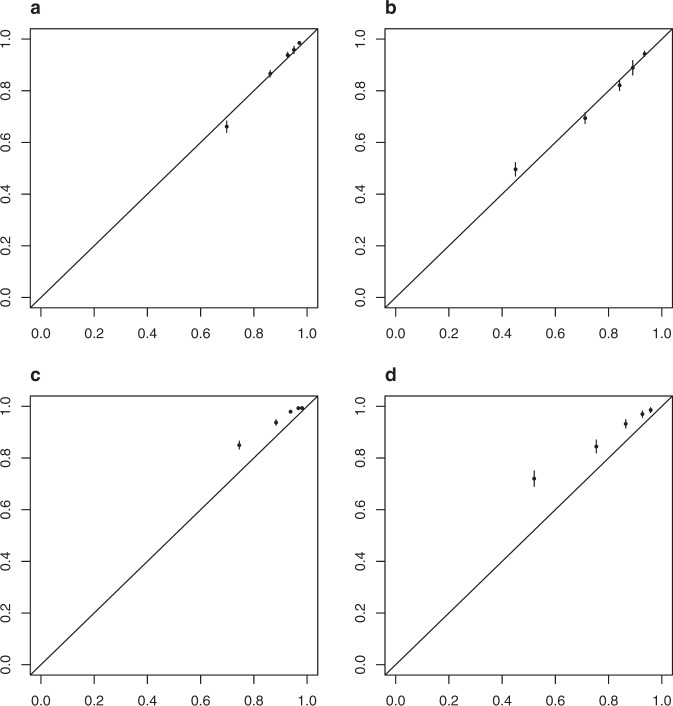
Calibration plots. **a** Training data, 5 years, **b** training data, 10 years, **c** validation data, 5 years and **d** validation data, 10 years.

**Fig. 4:**
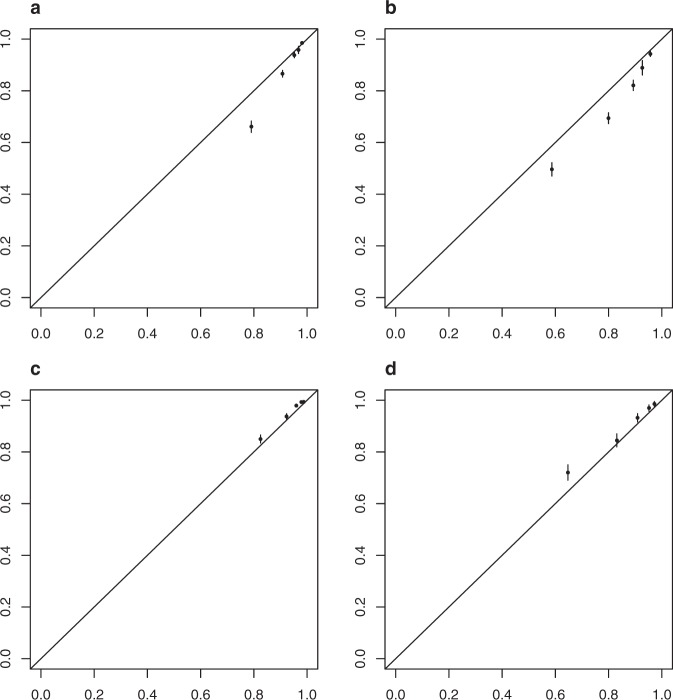
New calibration plots after recalibration. **a** Training data, 5 years, **b** training data, 10 years, **c** validation data, 5 years, and **d** validation data, 10 years.

Table 2 shows the patient characteristics for the 29,727 patients in the validation data set. The median follow-up was 2.4 years and the median age was 58 years. The 5-year BCSS estimate and 5-year OS was 95% and 91%, respectively, for the validation cohort. Outcomes were as follows: 3% BC-related mortality, 6% mortality from any cause, 1% locoregional recurrence, and 4% distant recurrence. BCSS at 5 years was 89% in the training set and 95% in the validation set while at 10 years BCSS was 78% in the training set and 89% in the validation set. Figure 2a, b show BCSS predictions in the training and validation sets stratified by risk set (based on the same centiles used for the training set). The c-index from the validation set is 0.82. Taken together, these indicate that the selected model discriminated well when applied to the validation set. However, Fig. 3a and b shows that our model is poorly calibrated when applied to the validation set. The predicted probabilities were much lower than the observed probabilities. Figure 4 shows that after recalibration, the predictions showed good calibration in the validation data.

## Discussion

In this analysis, we demonstrate statistical significance and improved predictive accuracy of incorporating age, grade, and receptor-based subtypes conditional on anatomic stage to improve prediction of breast cancer-specific survival (BCSS) using a Fine–Gray Model. In doing so, we have shown through a different statistical methodology and with a longer follow-up that more individualized prediction of BCSS is possible by considering clinical and biologic factors in addition to anatomic stage in a dataset with long followup that has been validated in an external dataset with demonstration of good calibration in both datasets. Calibration of the BCSS prediction model is evaluated by visual inspection of the 5-year and 10-year calibration plots, plotting the average predicted BCSS against the observed BCSS for each of 5 risk groups at 5 years and 10 years. The 5-year and 10-year BCSS of patients with particular values for the clinical and biological variables can be found using an online tool at the following link: http://mdanderson.org/BCSS.

We chose the Fine–Gray model to develop a BCSS prediction model over other potential and different statistical methods used in the existing online breast cancer survival prediction tools^35–38^. PREDICT, an online breast cancer prognostic and treatment benefit model, used a Cox proportional hazards model, which is frequently used in risk prediction models for breast cancer, to predict BCSS. When an individual in the risk set is exposed to more than one cause of failure (e.g., non-breast cancer death, which cannot be neglected for early stage breast cancers), resulting in competing risks, the Kaplan–Meier method of estimating CIF and the Cox regression model of estimating survival lead to biases^42^. The Fine–Gray model is a widely used method to build prediction models when competing risks exist (as is the case for early stage breast cancer). The Fine–Gray model allows estimation of the effect of the covariates on the CIF, but it has a limitation in its difficulty in interpreting the model coefficients, as compared to the Cox proportional hazards model^42–44^. That is, the model covariates can be interpreted as having an effect on the CIF, but they do not directly link to an underlying event rate in the real world^45,46^. This limitation, however, does not pose an issue in our analysis as the CIF is of equal clinical relevance in our study.

The results of our study should be interpreted with several considerations. First, the analysis was performed using data collected at a high volume single cancer center with specific referral and practice patterns leading to a potential selection bias. Our institution tends to treat larger and higher grade tumors with neoadjuvant systemic therapy, and the patient cohort analyzed in this study excluded those patients, possibly biasing the remaining group. One example of our referral bias is that the median age of patients in this database is lower than the national median age. However, since the incorporation of the other biomarkers had a similar effect in all age groups in this analysis, we consider that what we demonstrated in our database is likely generalizable to all age groups. Second, all patients did not have their surgery at our institution so there is the possibility of interobserver variability among pathologists. However, the majority of patients seen at MDACC have their pathology material reviewed by dedicated breast cancer pathologists, so this variability was likely largely reduced. Further, while inter-pathologist variability in determining grade has been amply documented in the literature, grade has always been a strong prognostic marker in all published analyses. Additionally, the majority of patients in the training dataset had only NG available. In a preliminary analysis not shown, concordance was evaluated among the 6150 patients in the training dataset with both NG and HG and 17,536 patients with both NG and HG in the validation dataset with a substantial or moderate agreement noted (Kappa 0.7 (training) and 0.56 (validation), respectively)^47^. Therefore, a decision was made to use NG interchangeably when HG was unavailable. Third, our results reflect heterogeneity of treatments selected by individual treating physicians, although the overwhelming majority would have received an anthracycline-based regimen, and most also received a taxane. Detailed adjuvant therapy was available in the training dataset but not available in the validation dataset. In the training dataset, we made a decision to exclude HER2+ patients who did not receive adjuvant therapy with trastuzumab-based regimens [HER2+(no T)] patients to provide survival probability estimates that better reflect outcomes of a contemporary population; however it has introduced a bias within this subset with respect to adjuvant treatment as well as time. Adjuvant treatment was not accounted for in the other biomarker subtypes and trastuzumab was not incorporated into standard adjuvant practice for HER2+ BC until 2005. Fourth, the data spanned over a large time period during which significant diagnostic and therapeutic advances have been made, resulting in a temporal cohort effect. Fifth, with respect to violations of proportional hazard, the hazard ratio changes over time making it challenging to represent this situation accurately with a single value (i.e., which is valid when the hazard ratio is constant over time). Thus, the estimate can be viewed as a weighted average of the hazard ratios which are changing over time. Future research will consider more complicated models, using time-varying covariate effects, in pursuit of more accurate prediction. Another consideration is that there is the potential for more complex interaction effects between these additional clinical and biological factors with other variables, such as adjuvant treatment and pathological stage, not accounted for in the analyses presented here. The median follow-up time in the training dataset is much longer than that of the validation dataset (8 years vs. 2.4 years) and this resulted in the need to recalibrate the predictions due to poor calibration of the final model when applied to validation data set. It is unclear why the final model did not fit the validation data well. However, a possible answer is the dissimilarity in the distributions of covariates and risk profiles between training and validation data sets. Finally, all deaths after breast cancer recurrence were considered as breast cancer-related deaths as the detailed death attributions are not available in our database.

Previously published work from MDACC has shown that a staging system that incorporates grade and HR status improves the disease-specific survival estimates as compared to anatomic stage alone^30^. In two recent presentations from MDACC, a novel risk score that includes grade, and ER, and HER2 status was evaluated in a contemporary patient population who received surgical intervention at MDACC and was then validated in a larger cancer registry confirming the importance of biologic factors in determining prognosis for breast cancer patients^48^. The MDACC database has also been supplemented with laboratory, patient lifestyle, and quality of life survey data showing further refinement in prognostic ability^49^. The recently updated AJCC 8th edition has recognized prognostic categories which are largely based on the NCDB analysis which contains over 300,000 women with invasive breast cancer diagnosed in 2010–2011 with a complete set of variables but short median follow-up (37.6 months). The inclusion of grade, and HER2, and HR status using the NCDB model resulted in stage reassignment for 41% of the patients to a stage group that was higher or lower than would otherwise have been assigned using the AJCC 7th edition anatomic stage categories^34^. Since the analyses confirming that prognosis varied within TNM groupings based on tumor biology and the identification of the new prognostic categories in the updated AJCC 8th edition, there have been several publications and validation papers further recognizing the importance of incorporating biological factors and confirming the effectiveness of the revised prognostic categories^50–53^.

While the breast cancer community has known for several decades the prognostic impact of grade, age, HR and HER2, no publication had shown that singly or in combination, these factors affected the outcomes of patients included in specific TNM stages. This analysis has clearly demonstrated the added prognostic value of patient and tumor characteristics when combined with anatomical stage. External validation confirmed discriminative ability of selected model and with recalibration the predictions were well calibrated to the validation data. In summary, we present the first user-friendly clinical tool developed to estimate BCSS based on an extensive analysis using Fine–Gray Model in a robust single institution database and validated in a nationally recognized-external database. The goal of developing this tool is to provide a resource for clinicians to help guide discussions with patients and provide an estimation of prognosis based on clinical and biological factors.

## Methods

### Training data patient population

A prospectively maintained electronic database of patients with breast cancer treated at The University of Texas MD Anderson Cancer Center (MDACC) was used to identify over 20,000 patients with stage I–III invasive unilateral breast cancer who received surgery as initial treatment from 1990 to 2016. Clinico-pathologic data was collected from the database, including age; stage; grade, estrogen receptor (ER), progesterone receptor (PR), and HER2 status; adjuvant treatment history; and disease status at the time of death. Pathological stage, tumor grade, HR status, and HER2 status were extracted from the surgical pathology report and determined according to AJCC guidelines^8,27,54–59^. For tumor grade, composite histologic grade was used primarily when available and if not available, then nuclear grade was used (Supplementary Text A). Disease status at the time of death and cause of death were ascertained for each patient. Patients were defined to have a breast cancer (BC)-related death if they died following a recurrence of breast cancer irrespective of the time elapsed between recurrence and death and the cause of death. Study data were collected and analyzed with approval from the Institutional Review Board (IRB) at the University of Texas MDACC. A waiver of consent was obtained due to the retrospective nature of the study. The data were transferred for analysis on April 5, 2016. Among the patients who were alive, 66% had a date of last follow-up within 2 years of April 2016. 58% of alive patients had follow-up longer than 5 years and 28% of alive patients had follow-up longer than 10 years.

### Validation data patient population

In the NCCN breast cancer outcomes database, a cohort of over 44,000 patients with pathological stage I–III unilateral primary invasive breast cancer who received surgery as an initial treatment from 1997 to 2012 were identified. Patients received all or some of their treatment at one of 16 NCCN participating centers between July 1st 1997 and December 31, 2012 (Supplementary Text B). Patients registered at the University of Texas MDACC (*n* = 7432) were excluded from the NCCN database to create an external dataset of 37,559 patients (non-MDACC NCCN cohort). Patients without complete data on age, anatomic stage, HR/HER2 status, and grade were excluded resulting in a validation cohort comprised of 29,727 patients who had complete data. All of the clinically relevant variables were obtained directly from the NCCN database. The last follow-up in validation data was 2/15/2013. Among patients who were alive, 79% had a date of last follow-up within 2 years of February 2013, 20% of patients alive had follow-up longer than 5 years, and 4% of patients alive had follow-up longer than 10 years.

### Statistical analysis

The primary endpoint was BCSS that was assessed while treating non-BC death as a competing risk. BCSS curves were estimated using the Aalen–Johansen method. Univariate and multivariate Fine–Gray proportional hazards models were fit to assess the statistical significance of the effects of the clinically relevant variables on BCSS. We coded HR/HER2 as a four-level categorical variable in all models. Harrell’s C-index was calculated to evaluate the discrimination capacity of each model^56^. The kmi package was used to impute censoring times for competing risk data and rcorr.cens in Hmisc package was used to estimate the C-index and its confidence interval. We checked the proportional hazards (PH) assumption by visually inspecting the smoothed, scaled Schoenfeld residuals and hazard ratios by time intervals, assessed nonlinear covariates effects using spline functions and checked for two-way covariate–covariate interactions by introducing product terms in the models. A *p*-value of <0.05 indicated statistical significance. Using a selected Fine–Gray model to estimate the BCSS probabilities (as 1−cumulative incidence function (CIF)) including patients with complete data, an online tool to estimate individual prognosis was developed. Prognostic index was defined as the weighted sum of the variables in the Fine–Gray model, where the weights were the regression coefficients. Model calibration was evaluated by comparing observed and predicted BCSS probabilities for five risk groups (defined by partitioning the prognostic index based on its 16th, 39th, 62nd, and 84th percentiles).

To assess the performance of our selected model on the validation data, we compute predictions for each patient in the validation set using the model fit to the training data and compare these predictions to the observed validation outcomes. Statistical analyses were performed using SAS 9.4 (SAS Institute Inc, Cary, NC). A more complete description of the statistical methods is given in the supplementary materials (Supplementary Text C).

## Supplementary information

Supplementary Table and Notes

## Data Availability

The data that support the findings of this study are available from the corresponding author, upon reasonable request. Please note that the NCCN data can only be made available with permission from NCCN.

## Code availability

Data management was performed in SAS 9.4; analyses were conducted using SAS 9.4, Tibco Spotfire S+8.2 for windows and R-3.6.1. Code is available upon reasonable request. Please contact the corresponding author.
