# Supplementary material for: Incorporation of clinical and biological factors improves prognostication and reflects contemporary clinical practice
Source: NPJ Breast Cancer. 2020 Mar 25;6:11. doi: 10.1038/s41523-020-0152-4 (PMC7096449; doi:10.1038/s41523-020-0152-4)
Supplement: Supplementary file 1 — Supplementary Table and Notes [file 41523_2020_152_MOESM1_ESM.pdf]

Supplementary Table 1. Patient characteristics for the overall training cohort, n = 20,928

Variable			N	%
Sex	Female		20753	99.0%
	Male		175	1.0%
Race	White		15381	74.0%
	Black		2047	10.0%
	Hispanic		2425	12.0%
	Other		1075	5.0%
Age at Diagnosis	< 40		2655	13.0%
	40-69		15898	76.0%
	≥70		2375	11.0%
Menopausal Status[1]	Premenopausal		7700	36.8%
	Postmenopausal		13228	63.2%
Anatomic Stage	I		10573	51.0%
	IIA		5509	26.0%
	IIB		2853	14.0%
	IIIAB		1464	7.0%
	IIIC		529	3.0%
Grade[2]	1		2254	10.8%
	2		8413	40.2%
	3		8838	42.2%
	Unknown		1423	6.8%
ER Status[3]	Positive		15072	72.0%
	Negative		4646	22.0%
	Unknown		1210	6.0%
PR status[3]	Positive		12519	60.0%
	Negative		6827	33.0%
	Unknown		1582	8.0%
Hormone Receptor (HR)	HR+[4]		15530	74.0%
	HR- [5]		4151	20.0%
	Unknown		1247	6.0%
HER2 Status[3]	Positive[6]		2312	11.0%
	Negative		14137	68.0%
	Unknown		4479	21.0%
Biomarker Subgroups	Triple Negative		2557	12.0%
	HR+/HER2–		11542	55.0%
	HR+/HER2+		1380	7.0%
	HR-/HER2+		912	4.0%
	Unknown		4537	22.0%
Adjuvant therapy received	Chemotherapy		11404	55.0%
		HR-/HER2- (n=2557)	2012	79%
		HR+/HER2- (n=11542)	5227	45%
		HER2+ (n=2312) [6]	1866	81%
		HR+/HER2+ (n=1380) [7]	1090	79%
		HR-/HER2+ (n=912) [7]	763	84%
	Endocrine therapy		13150	63.0%
	HER2 targeted therapy		1227	6.0%

KEY:

1. Clinically defined based on history; if not recorded, age is considered (≥ 60: postmenopausal; < 60: Premenopausal)
2. Histologic grade (HG) or nuclear grade (NG) (if HG is not available). For the 19,505 patients with known grade: HG (n=6353) and NG (n=13152).
3. ER: estrogen receptor; PR: progesterone receptor; HER2: HER2-neu receptor; Biomarker definitions in database are reflective of evolution of national practice guidelines.[28,38-41]
4. HR+: ER+ or PR+
5. HR-: ER- and PR-
6. 53% received adjuvant trastuzumab (T); 20 patients have unknown HR status
7. 54% of HR+/HER2+ and 63% of HR-/HER2+ patients received adjuvant trastuzumab (T)

Supplement Methods
Supplementary Text A.

For patients who had surgery outside of the institution, if MDACC pathology review was unavailable, then the outside surgical pathology report was used for data extraction.

Anatomic stage: In an initial analysis, the Aalen-Johansen estimates for stage IA and stage IB were overlapping indicating a ≥95% 5-year BCSS irrespective of tumor size; therefore, these patients were combined into one group referred to as "Stage I". Additionally, due to the small number of patients in stage IIIB, these patients were combined into a stage IIIAB group.

Tumor grade: In defining a composite tumor grade, we used the histologic grade (HG) when it was available. For patients without HG available, the nuclear grade (NG) was used. The majority of patients in the training set had NG and the majority of patients in the validation set had HG. An initial analysis showed that HG and NG had a relatively good concordance. Specifically, among patients whose HG and NG were both available, the Kappa was 0.7 (weighted Kappa=0.73) for the training set and 0.56 (weighted Kappa=0.58) for the validation set. These values represent a substantial and moderate agreement between HG and NG in the training and validation sets, respectively.[1] The Spearman correlation coefficient was 0.80 and 0.68 for the training set and the validation set, respectively. A different composite tumor grade variable was considered by dividing each grade into 2 subgroups, depending on the availability of HG. Six groups were formed as grade 1 by HG (HG=1), grade 1 by NG (no HG and NG=1), grade 2 by HG (HG=2), grade 2 by NG (no HG and NG=2), grade 3 by HG (HG=3), and grade 3 by NG (no HG and NG=3). The prognostic accuracy measured by C-index was comparable (improvement was only 0.001 between these two definitions) when using the tumor grade (with three levels) versus using the composite tumor grade (with six groups) in multivariate Fine-Gray models including other covariates (stage, HR/Her2, tumor size, age, race, and number of nodes positive).

HR status: HR+ was defined as either ER positive (ER+) or PR positive (PR+). HR negative (HR-) was defined as ER- and PR-. The data were collected as ER- or PR- if less than 10% until 2010 and <1% after 2010. [2]

HER2 status: Testing for HER2 has been refined over time to reflect the predictive and prognostic value of HER2 status. [3-6] Given the impact of adjuvant trastuzumab-based therapy on outcomes for HER2+ breast cancer, we identified patients who received adjuvant trastuzumab (HER2+ (T)) and those who did not (HER2+ (no T)). Additionally, patients were documented as HER2+ (no T) if the receipt of trastuzumab was unknown or if they were treated prior to the approval of adjuvant trastuzumab in the non-metastatic setting in 2005. Prior to 2006, HER2 status was evaluated by immunohistochemistry and/or by FISH (Fluorescent in situ hybridization) and determined based on the contemporary testing practices.[3,4] After adjuvant trastuzumab became the standard of care

in 2006, HER2 status was determined as positive if there was overexpression by IHC (3+) or FISH amplification (HER2 and chromosome 17 ratio equal or higher than 2.2 or average HER2 gene copy number greater than 6.0).[5] In 2013 a change was instituted reflecting the updated ASCO/CAP guidelines and the ratio cutoff defining positivity moved to back to 2.0.[6] If FISH was not available or not performed, IHC 2+ was considered an unknown result. Similarly, if neither was confirmed or available, the patient's HER2 status was documented as unknown. Patients were determined to have a negative HER2 status as follows: IHC 0-1+ and/or FISH-, IHC 2+ and FISH-.

Supplementary Text B

The following are the 16 NCCN participating Centers: City of Hope Comprehensive Cancer Center, Dana-Farber Cancer Institute/Brigham and Women's cancer center/Massachusetts General Hospital Cancer Center, Fox Chase Cancer Center, The University of Texas MD Anderson Cancer Center (MDACC), Roswell Park Cancer Institute, University of Michigan Comprehensive Cancer Center, The Ohio State University Comprehensive Cancer Center, Moffitt Cancer Center, The Fred & Pamela Buffett Cancer Center at University of Nebraska Medical Center, Duke Cancer Institute, Robert H. Lurie Comprehensive Cancer Center of Northwestern University, The Sidney Kimmel Comprehensive Cancer Center, Fred Hutchinson Cancer Research Center, Huntsman Cancer Institute, Siteman Cancer Center, UCSF Helen Diller Family Comprehensive Cancer Center). Patients received their care at the participating institution for at least 365 days after their first visit date. The institutional review boards at each center approved the protocol, data collection processes, data transmission methods, and data repository protocols.

Supplementary Text C

Clinical Endpoints

The main outcome of interest was breast cancer-specific survival (BCSS), defined as the time from the date of surgery to date of BC-related mortality (death with disease). For patients who died without having experienced a recurrence of breast cancer, time from surgery to date of non-BC related mortality was calculated. Patients without a documented date of death were censored at the last follow-up. Breast cancer specific mortality is an event of interest. Non-BC related mortality is a competing risk event.

Training Data

Patient demographical and disease characteristics were summarized by means (standard deviations) and frequency (%). Univariate and multivariate Fine-Gray proportional hazards models were fit to assess the statistical significance of the effects of the clinically relevant variables on BCSS both univariately and when controlling for the other factors, respectively. Specifically, multiple models were fit with various combinations of factors taking into account situations where limited information might be available. A cohort of patients with complete data on age, HER2, ER, PR, grade and anatomic stage was used for model comparison purposes. Harrell's C-index was also calculated to evaluate the predictive power of each model. We selected the most clinically applicable model by focusing more on the practical availability of information from these factors while

accommodating preferred models based on the Harrell's C, with models with larger Harrell's C being preferred. The Aalen-Johansen estimator and a selected Fine-Gray model based on both considerations on availability of information in clinical practice and the above model selection/assessment criteria were used to estimate the BCSS probabilities (as 1 minus cumulative incidence function (CIF)) including all patients with complete data on selected factors and based on the selected model an online tool to estimate individual prognosis has been developed. We also fit multiple models with various combination of factors, including all patients with complete data on each combination. We checked proportional hazards assumption using Schoenfeld residuals, assessed nonlinear covariates effects using spline function and checked for two-way covariate-covariate interaction by introducing product terms in the model. A p-value of less than 0.05 indicated statistical significance. Statistical analyses were performed using SAS 9.4 (SAS Institute Inc, Cary, NC).

Validation Data

To assess the performance of our selected model on the validation data, we compute predictions for each patient in the validation set using the model fit to the training data and compare these predictions to the observed validation outcomes. Specifically, BCSS, as 1 minus CIF, with CIF being estimated by the Aalen-Johansen estimator in the validation set stratified by risk set (partitioned by the 16th, 39th, 62nd, and 84th percentiles), assessed discrimination ability of the selected model, and calibration of the model was evaluated by comparing observed and predicted BCSS probabilities for 5 risk groups. To create risk groups, we categorized the prognostic index (PI) into 5 groups at the 16th, 39th, 62nd, and 84th percentiles, giving 2 smaller groups at relatively low and high risks of breast cancer-specific death, respectively, and three larger, central groups at lower or higher intermediate risks. On a standard normal scale, the 39th and 62nd percentiles correspond to approximately +/-1SD from the mean. Calibration plots at 5-years and at 10-years were generated by plotting the average predicted BCSS against the observed BCSS for each risk group at 5-years and 10-years and the 45 degree line representing ideal calibration. The prediction model was recalibrated by updating the model intercept (corresponding to the baseline BCSS estimate) with or without updating the regression coefficient of the prognostic index (PI) in the validation data to account for a different baseline BCSS function in the validation data and/or to account for a different regression coefficient of the PI in the validation data. The recalibrated survival was obtained as $S_{0,new}(t)^{exp(PI)}$ or $S_{0,new}(t)^{exp(\beta * PI)}$, where $S_{0,new}(t)$ was the recalibrated baseline BCSS function and β was the calibration slope, i.e., the slope obtained from the Fine-Gray model with the PI as the only predictor. Four different recalibrations were considered: 1) using the baseline validation BCSS estimate without a recalibrated slope; 2) using the average of the baseline BCSS estimates from the training and validation data without a recalibrated slope; 3) using the baseline validation BCSS estimate with a recalibrated slope; and 4) using the average of the baseline BCSS estimates from the training and validation data with a recalibrated slope. Recalibration with the average of the baseline BCSS from the training and validation data without a recalibrated slope was selected, because it provides a reasonable calibration for the training data set and more importantly, a good calibration for the validation set. The root mean square prediction

error at 5 years and 10 years showed that using the average of the baseline survivals from the training and validation sets yields a reasonable calibration.

The predictions after re-calibration were compared with the observed survival probabilities to evaluate the calibration aspect of the recalibrated predictions. The recalibration was presumably necessary because of the significant differences in BCSS between the training and validation data (even after covariate adjustments).

Development of online tool

To facilitate use in the clinical setting, we have developed an online interface through which physicians may input the variables of interest (age, pathologic stage, tumor grade, ER, PR, and HER2) and receive an output of the estimated 5-year and 10-year BCSS to share with their patients based on the analysis provided in this manuscript. The tool currently requires input of all variables. However, we recognize that in some clinical settings all variables may not be available and therefore, we intend to update this tool with the option to receive an estimate even with missing variables.

References

1. McHugh ML: Interrater reliability: the kappa statistic. Biochem Med (Zagreb) 22:276-82, 2012
2. Hammond ME, Hayes DF, Dowsett M, et al: American Society of Clinical Oncology/College Of American Pathologists guideline recommendations for immunohistochemical testing of estrogen and progesterone receptors in breast cancer. J Clin Oncol 28:2784-95, 2010
3. Slamon DJ, Leyland-Jones B, Shak S, et al: Use of chemotherapy plus a monoclonal antibody against HER2 for metastatic breast cancer that overexpresses HER2. N Engl J Med 344:783-92, 2001
4. Romond EH, Perez EA, Bryant J, et al: Trastuzumab plus adjuvant chemotherapy for operable HER2-positive breast cancer. N Engl J Med 353:1673-84, 2005
5. Carlson RW, Moench SJ, Hammond ME, et al: HER2 testing in breast cancer: NCCN Task Force report and recommendations. J Natl Compr Canc Netw 4 Suppl 3:S1-22; quiz S23-4, 2006
6. Wolff AC, Hammond ME, Hicks DG, et al: Recommendations for human epidermal growth factor receptor 2 testing in breast cancer: American Society of Clinical Oncology/College of American Pathologists clinical practice guideline update. J Clin Oncol 31:3997-4013, 2013

6\. Wessler BS, Ruthazer R, et al: Regional Validation and Recalibration of Clinical Predictive Models for Patients With Acute Heart Failure. J Am Heart Assoc. 2017 Nov 18;6(11)

7\. Steyerberg EW, Vergouwe Y: Towards better clinical prediction models: seven steps for development and an ABCD for validation. Eur Heart J. 2014 Aug 1;35(29):1925-31

8. Steyerberg EW. Clinical Prediction Models. New York, NY: Springer New York; 2009
